# Magnetic torque anomaly in the quantum limit of Weyl semimetals

**DOI:** 10.1038/ncomms12492

**Published:** 2016-08-22

**Authors:** Philip J. W. Moll, Andrew C. Potter, Nityan L. Nair, B. J. Ramshaw, K. A. Modic, Scott Riggs, Bin Zeng, Nirmal J. Ghimire, Eric D. Bauer, Robert Kealhofer, Filip Ronning, James G. Analytis

**Affiliations:** 1 Department of Physics, University of California Berkeley, 359 Birge Hall, Berkeley, California 94720, USA; 2 Max-Planck-Institute for Chemical Physics of Solids, Nöthnitzer Strasse 40, Dresden 01187, Germany; 3 National High Magnetic Field Laboratory, TA-35, Los Alamos, New Mexico 87545, USA; 4 National High Magnetic Field Laboratory, 1800 E. Paul Dirac Dr., Tallahassee, Florida 32310, USA; 5 Los Alamos National Laboratory, TA-3, Los Alamos, New Mexico 87545, USA

## Abstract

Electrons in materials with linear dispersion behave as massless Weyl- or Dirac-quasiparticles, and continue to intrigue due to their close resemblance to elusive ultra-relativistic particles as well as their potential for future electronics. Yet the experimental signatures of Weyl-fermions are often subtle and indirect, in particular if they coexist with conventional, massive quasiparticles. Here we show a pronounced anomaly in the magnetic torque of the Weyl semimetal NbAs upon entering the quantum limit state in high magnetic fields. The torque changes sign in the quantum limit, signalling a reversal of the magnetic anisotropy that can be directly attributed to the topological nature of the Weyl electrons. Our results establish that anomalous quantum limit torque measurements provide a direct experimental method to identify and distinguish Weyl and Dirac systems.

## Abstract (web summary)

The topological character of electrons in semimetals subtly influences their bulk properties, leading typically to weak experimental signatures. Here, Moll *et al*. report a distinctive anomaly in the magnetic torque upon entering quantum limit state in the Weyl semimetal NbAs, which only appears due to the presence of Weyl fermions.

## Graphical abstract

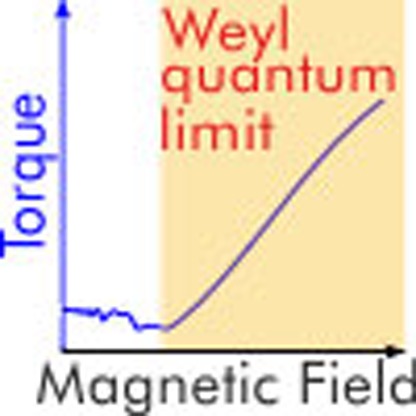

Quasiparticles with linear dispersion in condensed matter^1^ systems have sparked considerable excitement since their discovery in graphene^2^, topological insulators^3^, Dirac semimetals such as Na_3_Bi^4^,^5^, Cd_3_As_2_ (refs 6, 7) and the mono-pnictide class of Weyl semimetals (Ta,Nb)(As,P)^8^,^9^. The research interest is fuelled by the technological potential of topological materials in devices exploiting the relativistic nature of the electrons^10^,^11^,^12^, such as ultra-high mobilities, the topological protection from back-scattering and the massless behaviour of charge carriers. Given the wealth of novel phenomena observable in these materials^13^, it is important to find clear experimental signatures that can help identify new compounds as topological semimetals. Band structure calculations can point towards new materials with non-trivial band topology. However, due to the relatively small spin-orbit energy scale, the presence of topological charge carriers at the Fermi level is very sensitive to subtle shifts in the chemical potential. For example, band structure calculations and quantum oscillation experiments on the candidate Weyl semimetal TaP have recently shown a Weyl point just ∼15 meV above the band bottom thus challenging the topological character of this material^14^. Hence, clear and simple experimental tools for determining the topological character of the quasiparticles are highly desirable to accelerate the search for materials.

Here we demonstrate that magnetic torque can distinguish between topological and trivial electrons. A key aspect of our study is that this probe can also experimentally distinguish Weyl points from closely related Dirac points—which consist of two coinciding Weyl cones protected by crystal symmetry. Such a distinction is not possible with spectroscopic techniques operating at magnetic fields well below the quantum limit, such as angle-resolved photoemission or scanning tunnelling spectroscopy, which are only sensitive to the electronic dispersion and not its topology. We show that the non-zero Berry's phase in Weyl semimetals manifests itself as a magnetic torque anomaly at the quantum limit, where all electrons in a band are confined to the last Landau level (LL).

Strong magnetic fields, B, quantize the motion of electrons with an effective cyclotron mass *m*_eff_ and a Fermi velocity *v*, onto LLs of energy *ɛ*_*n,k*_. The effect of a magnetic field on the energy spectrum in each of the three cases is


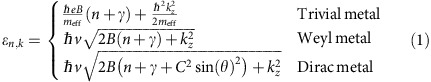


where *e* denotes the electron charge, *n* the LL index, *k*_*z*_ the momentum component along the magnetic field *B*, *θ* the angle between the field and the line connecting the Dirac nodes in momentum space and *C* a material-dependent parameter encoding the details of the spin-orbit coupling on the band structure. In trivial metals, the quantum correction term *γ* takes the value 1/2 (ref. 15), but in Weyl and Dirac systems it attains a contribution known as Berry's phase^16^,^17^, such that *γ*=0. This topological property therefore depends only on the existence of separate Weyl nodes and not on the details of the band structure.

This energy relation (equation (1)) highlights the important differences between the different topologies in the quantum limit (*n*=0). In a trivial metal, the zeroth LL continues to increase linearly in energy with increasing magnetic field. In Weyl metals, the energy of the zeroth level is independent of the magnetic field. On the other hand, in Dirac metals the field allows the degenerate Weyl electrons to interact, opening a gap that depends on the direction and magnitude of the applied field. Therefore the zeroth Landau level of Weyl-semimetals remains field-independent for fields along all directions (*n*, *γ*=0), while those in trivial and Dirac systems are in general field-dependent.

The field dependence of the *n*=0 LL can be directly probed by measurements of the magnetic torque *τ*=*M*(*H*) × *H*, where 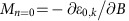. The magnetization per electron at zero temperature in the quantum limit in trivial metals saturates to a field-independent value 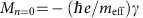, that of a Dirac system becomes strongly angle dependent as 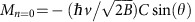, yet in the Weyl case the magnetization of the conduction electrons vanishes, *M*_*n*=0_=0. The saturation of magnetization without any magnetic anomaly at the quantum limit in topologically trivial conductors is well understood and commonly observed in low carrier density semiconductors where the quantum limit can be easily accessed, for example InSb and InAs^18^. In Weyl metals, however, the collapse of the magnetization leads to a magnetic anomaly^19^,^20^. Similar arguments have explained the anomalous magnetization of LaRhIn_5_ in the quantum limit, where a different type of topological defect (band-crossing line) generates Berry's flux^21^,^22^,^23^.

We now turn to the experimental evidence for the torque anomaly at the quantum limit of the Weyl semimetal NbAs that can be quantitatively understood based on the topological character of the electronic dispersion, without invoking details of the band structure.

## Results

### Anomalous torque in the quantum limit

NbAs was chosen as a Weyl semimetal to search for experimental signatures of the proposed magnetic anomaly^24^. The small Fermi surfaces of this material allow us to reach and exceed the quantum limit for a large angle range in static magnetic fields up to 45 T and pulsed magnetic fields up to 65 T. Similar to the recently investigated related phosphides NbP and TaAs, NbAs is expected to host both trivial electrons and Weyl fermions on different Fermi surface sheets^8^,^25^. We have measured resistance and magnetic torque of single crystal NbAs using both capacitive and piezo-resistive cantilever torque techniques in pulsed and dc-fields, and find quantitative agreement between different samples and techniques (see Methods).

**Figure 1:**
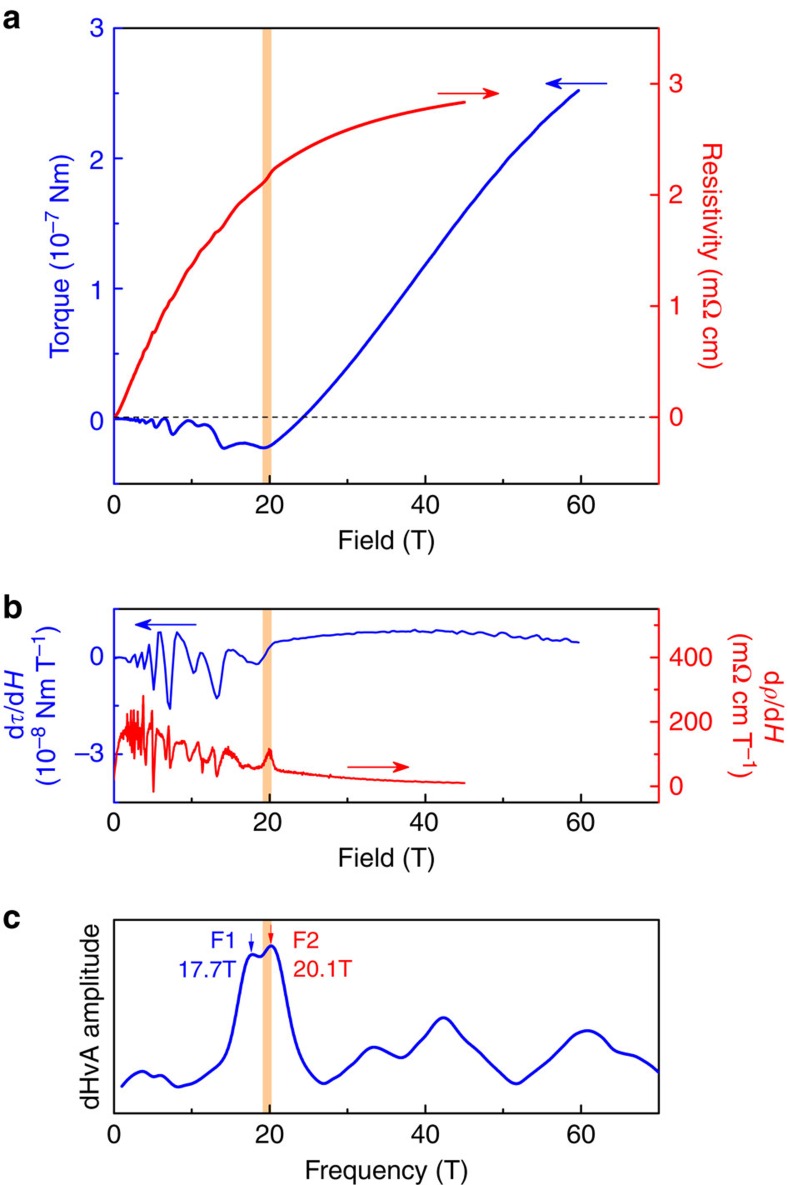
Magnetic torque and resistivity of NbAs across the quantum limit. These data were measured at a temperature of 4 K and at an angle of 25° off the c-axis towards the a-axis. (**a**) The low-field torque follows the conventional quadratic behaviour and shows strong quantum oscillations up to the quantum limit (orange line). Above the quantum limit, the torque grows strongly in magnitude, signalling the loss of the Berry-paramagnetic component beyond the quantum limit. In contrast to the torque, there is no change in behaviour in the smoothly saturating magnetoresistance beyond the last quantum oscillation. The torque was measured in pulsed fields up to 65 T, and resistivity in dc-fields up to 45 T. The quantum limit field is self-consistently determined as the position of the last quantum oscillations (**b**) and via the de Haas-van Alphen frequency (**c**) indicating the two close-by extremal orbits F1 and F2.

Figure 1 contrasts the torque and resistivity of NbAs in high fields, highlighting the main experimental observation: at the quantum limit, the magnetic torque shows a well-defined kink and a cross-over to a sizable, almost linear increase with a smooth change in slope up to the highest fields of 65 T. The position of the break in slope in the torque around 20 T (for fields 25° off the c-axis towards the a-axis) matches well with the observed extremal Fermi surface cross-section from the low-field quantum oscillation measurements and the position of the last quantum oscillation of the resistivity and torque, providing independent confirmation that the torque anomaly coincides with the quantum limit. Two close-by frequencies, F1 and F2, are observed in NbAs, in agreement with band structure calculations predicting the presence of both trivial and non-trivial Fermi surface sheets^26^. The limited field window and the low frequencies prevent a clear disambiguation of the Fermi surface responsible for the torque anomaly from our data, yet a previous quantum oscillation study associated the smaller frequency, F1, with a non-zero Berry's phase. However, the torque anomaly clearly confirms the presence of Weyl fermions in NbAs^26^.

The transverse magnetoresistance is very large, similar to recent reports in other topological metals^27^, reaching a 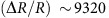 from *ρ*(0*T*)=0.3 *μ* Ω cm to *ρ*(45*T*)=2833.3 *μ* Ω cm. Yet the smoothly saturating resistivity remains featureless as the system crosses the quantum limit, in contrast to the torque.

### Angular dependence of the quantum limit

**Figure 2:**
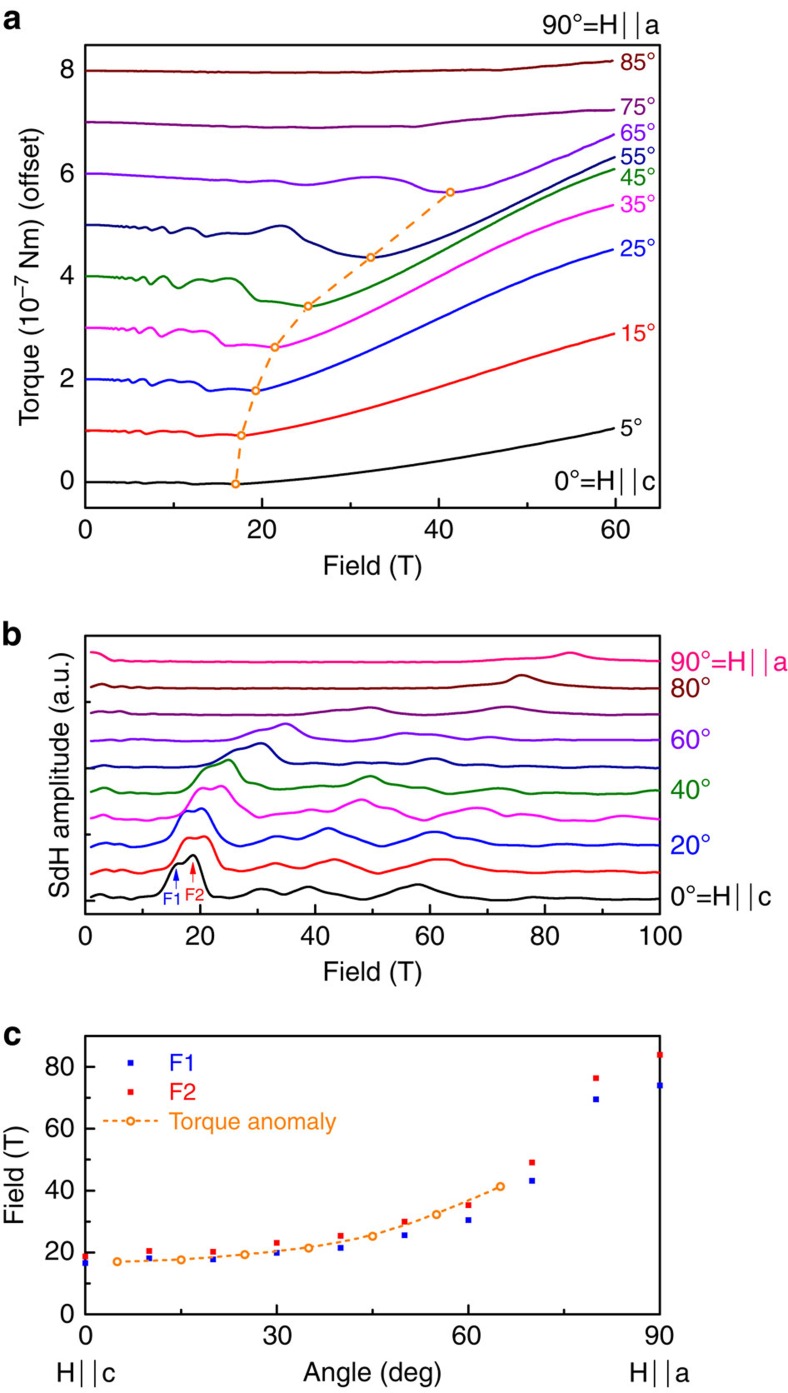
NbAs magnetic anomaly. (**a**) Angle dependence of the torque in high magnetic fields at 4 K, tilting the field from the c-direction (0°) towards the a-direction (90°). A clear break in slope at the quantum limit is observed in all traces up to 65°. (**b**) The quantum limit as a function of field angle was determined by low-field Shubnikov-de Haas oscillations. The oscillations contain a large harmonic contribution, leading to pronounced higher harmonics in the FFT spectrum. (**c**) The quantum limit tracks well the torque anomaly at all angles where it is experimentally accessible.

The Fermi surface anisotropy leads to an angle dependence of the quantum limit field (Fig. 2). The extremal cross-sections of the Fermi surfaces grow from 16 T for fields aligned with the crystal c-axis to 80 T as the field is tilted towards the a-axis. The position of the kink in the magnetic torque is found to coincide with the quantum limit field obtained by dHvA frequencies over the whole angle range up to 65° (Fig. 2c), above which the quantum limit exceeds the maximal fields available in this experiment of 65 T. The break in slope of the magnetic torque thus is clearly associated with the transition into the quantum limit (*n*=0) over the entire experimentally accessible angle range.

### Signatures of Berry paramagnetism

**Figure 3:**
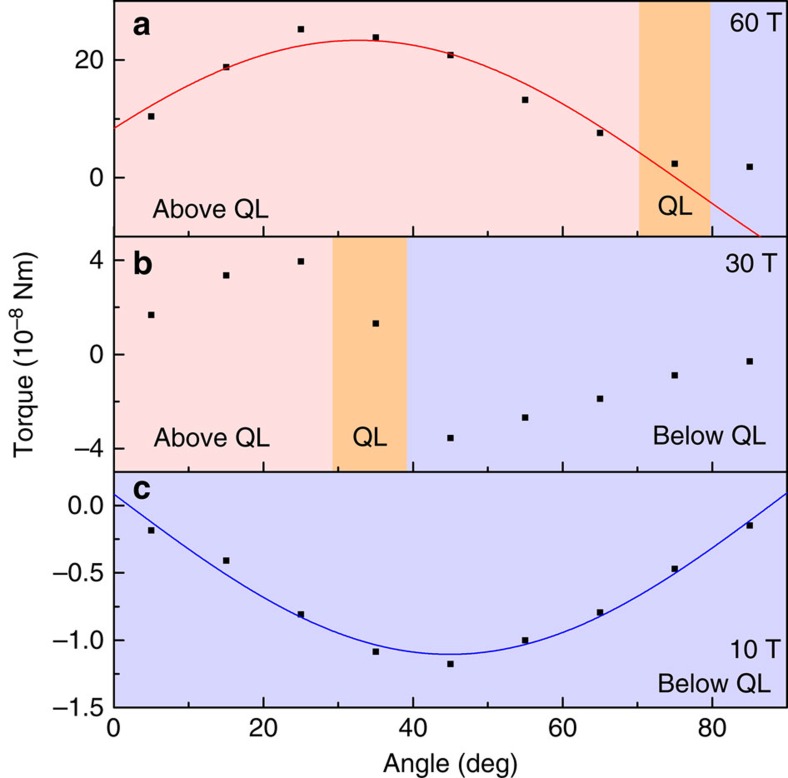
Magnetic anisotropy reversal at the quantum limit. The angle dependence of the non-oscillatory torque background at three different field levels: (**a**) In the high-field regime at 60 T above the quantum limit (QL) for most angles, (**b**) at 30 T in an intermediate regime, and (**c**) at 10 T below the QL. The torque in the low- and high-field regions both follow a sin(2*θ*) dependence, yet of opposite sign. This change in the magnetic anisotropy is most evident in the intermediate field region, where the system crosses the quantum limit as a function of angle. At low angles, the system follows the high-field anisotropy, then suddenly transitions into the low-field anisotropy at high angles where the quantum limit exceeds 30 T, leading to pronounced deviations from the usual sin(2*θ*) behaviour. Small deviations from the sign reversal between the 10 T and 60 T torque may appear due to the eventual cross-over into the low-field regime even at 60 T at high angles, and the field dependence of the susceptibility.

The sudden reversal of the slope of the magnetic torque at the quantum limit is a hallmark of Weyl fermions. In NbAs, the magnetic torque anomaly dominates the magnetic response and leads to unusual behaviour: Above the quantum limit, the torque changes sign, indicating a reversal of the magnetic anisotropy driven by the conduction electrons. This sign change occurs in the smoothly evolving, non-oscillatory component (see Methods for details on the background extraction). Figure 3 shows the angle dependence of the non-oscillatory torque at fixed fields, in the high-field (60 T) and low-field (10 T) region as well as an intermediate field value (30 T). Both the high- and low-field torque follow a sin(2*θ*) dependence, as expected for metals without permanent magnetic moments. The sign of the torque, however, is opposite below and above the quantum limit. This reversal occurs directly at the quantum limit, as evidenced by the strong deviation from sin(2*θ*) of the torque at intermediate fields. At low angles close to the c-axis and fields of 30 T, the system is above the quantum limit (compare with Fig. 2). As the field is rotated towards the a-axis, the torque increases tracking the high-field behaviour. At the same time the quantum limit field increases upon tilting the field towards the a-axis due to the anisotropy of the Fermi surface, and reaches 30 T at around 45°. At this point, the system crosses into the low-field state as the quantum limit is pushed above 30 T, which is accompanied by a sudden drop of the torque crossing through zero and reaching the same amplitude on the negative side. As the angle is increased further, the torque now tracks the low-field behaviour.

## Discussion

**Figure 4:**
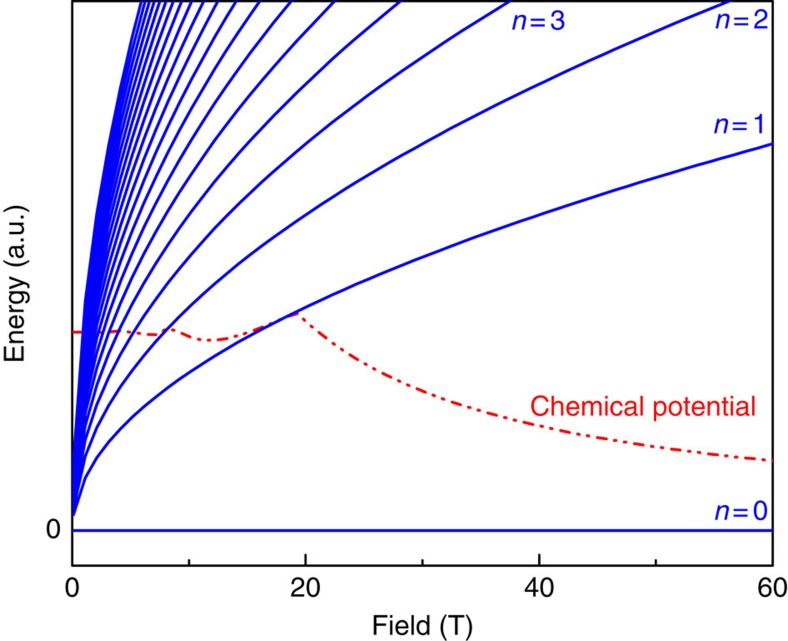
Berry paramagnetism. Field dependence of the Landau levels and the chemical potential in a Weyl-semimetal. The average chemical potential shrinks with increasing magnetic field, thus contributing a paramagnetic response.

This peculiar magnetic response can be understood as a sudden loss of balance of competing magnetic responses at the quantum limit. Weyl fermions in states above the Weyl node contribute a paramagnetic response, while those of the states below the node are diamagnetic. The origin of this paramagnetism is the presence of a field-independent zeroth LL in topological semimetals (equation (1)). The growing degeneracy factor pulls the chemical potential towards zero with increasing field, and thus causes a paramagnetic response as 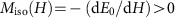 (Fig. 4). This unusual band paramagnetism is rooted in the *π* Berry phase of these materials and the resulting field-independent zeroth LL; hence we refer to it as Berry paramagnetism.

**Figure 5:**
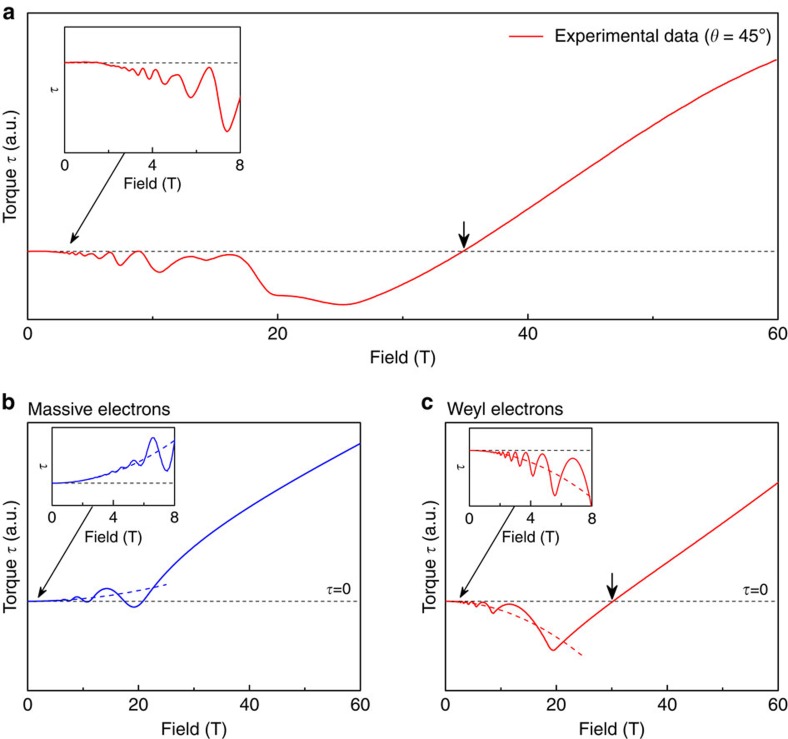
Comparison to calculated high-field torque. (**a**) Measured torque in NbAs at *θ*=45°. At low fields, the torque is negative and a clear turn in slope is observed at the quantum limit leading eventually to the sign change at higher field. This is compared to the theoretical expectation from equation (2) using the dispersion of massive (**b**) and Weyl (**c**) electrons. While for massive electrons a monotonous non-oscillatory torque is expected to transition smoothly through the quantum limit, the non-trivial topology in Weyl systems leads to a strong break-in-slope at the quantum limit. The only free parameters are the absolute magnitude of the susceptibility, and the momentum cutoff Λ∼1 Å^−1^. Importantly, this calculation is based on an ellipsoidal Fermi surface and nonetheless captures the essential physics at the quantum limit which is dominated by the topological character of the bands. The complex band structure of NbAs modifies the details, such as the presence of beating of multiple quantum oscillation frequencies.

At low fields, these two competing effects nearly cancel in NbAs, yet at the quantum limit the magnetism of the states at positive energy is quenched as all states are forced into a field-independent ground state. To model this response, we begin by computing the magnetization for an idealized isotropic Weyl node: 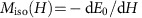, where 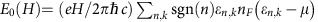 is the ground state energy, and the Fermi-function *n*_*F*_ restricts the sum to occupied orbitals (see Methods for further details on the calculation). The chemical potential, *μ*, is determined by the total particle density, *ρ*, (measured relative filling up to the Weyl node) via the relation: 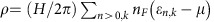. The states below the Weyl node (*n*<0) are far from the quantum limit and contribute a non-oscillatory diamagnetic response 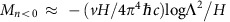, where Λ is the characteristic momentum at which the dispersion deviates from its linear form. The Berry paramagnetism dies away as the occupied conduction states are subsumed into the field-independent *n*=0 level. The residual diamagnetic contribution beyond the quantum limit comes from the valence band electrons, and will be enhanced by additional pockets of massive non-relativistic electrons, as in the case of NbAs (Fig. 5). To model the torque measurements, the results of this isotropic model can be related to the more realistic case with anisotropic velocities along the c- and a,b-axes, *v*_*c*_=*λv*_*a,b*_ by a simple rescaling:


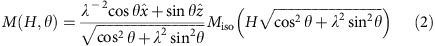


Comparing the quantum-limit field for *θ*=0°, 90° from the quantum oscillation spectrum (Fig. 2c) yields an anisotropy parameter *λ*≈0.4.

The torque reversal at the quantum limit is a direct consequence of the Berry's flux contained in the quasiparticle orbits (*γ*=0) and does not depend on the details of the band-structure. Trivial electrons always show the same magnetic response above and below the quantum limit, and therefore no magnetic anomaly is expected nor experimentally observed. The complicated angle dependence of the energy spectrum of Dirac materials (equation (1)) yields a strongly non-sinusoidal torque response. A magnetic torque following the conventional sin(2*θ*) functional form but of opposite sign compared to the low field state is thus a distinctive signature of Weyl fermions. Therefore, torque measurements in the quantum limit can uniquely distinguish experimentally between Weyl- and Dirac fermions. This may prove useful in the future search for topological materials as intrinsic (for example, vacancy ordering) or extrinsic (for example, strain) effects may break the crystal symmetry protecting the degeneracy of the Dirac dispersion, thus transforming massless Dirac fermions into Weyl or massive Dirac fermions. Further details on the differences between the torque responses of Weyl and Dirac semimetals are given in the Methods section.

Materials with a significant fraction of Weyl electrons at the Fermi level are most interesting for both applications as well as for the study of topological semimetals. In these materials the Weyl electrons dominate the magnetic response and the sign reversal is expected to be most prominent. However, even if the background magnetization is dominated by magnetic order or strong diamagnetism from large numbers of trivial electrons, the quantum limit torque anomaly will still appear as a change in slope on top of this background. The topological semimetals of interest for potential applications generally have small Fermi surfaces to avoid non-linear corrections to the band structure far away from the nodes, and thus the quantum limit can be easily accessed. High-field torque measurements thus provide a robust, simple and effective experimental tool to identify new topological materials, and to distinguish experimentally between Weyl- and Dirac fermions.

## Methods

### Crystal synthesis

NbAs single crystals were grown by chemical vapour transport using iodine as the transport agent. First, NbAs powder was prepared by heating a stoichiometric mixture of Nb powder and As pieces sealed in a quartz tube under vacuum. The ampule was slowly heated up to 700 °C and kept at this temperature for 3 days. Then 2 g of NbAs powder was mixed with 0.5 g of iodine and sealed in a quartz tube. The sealed ampule was loaded into a horizontal tube furnace for 10 days. The temperature of the hot end was maintained at 950 °C and that of the cold zone was approximately 850 °C. Several well-facetted crystals were obtained inside the quartz tube. The crystal structure was verified using room temperature X-ray diffraction.

### Sample characterization

The samples used in this study were obtained from the same growth batch, which also produced the crystals used in previous studies^24^,^26^. Samples from this growth were characterized by energy dispersive X-ray spectroscopy, Laue, powder and single crystal X-ray diffraction. As reported previously, the EDS suggests an off-stoichiometry of 6% excess Nb, which however is close to the expected uncertainty of the measurement^24^. Refinement of the single crystal diffraction data gave a goodness of fit on *F*^2^ of 1.243 with lattice parameters *a*=*b*=0.34516(13) nm and *c*=1.1672(5) nm using the NbAs structure type I41md (109). This is in good agreement with the published literature values, and suggests a stoichiometric sample^28^. The high crystallinity is further substantiated by mobilities in excess of 5 × 10^6^ cm^2^ V^−1^ s^−1^, and large Shubnikov de Haas oscillations, which were reproduced on multiple samples.

### Torque measurements

The magnetic torque was measured at the National High Magnetic Field Laboratory using CuBe cantilevers with capacitive readout in steady fields up to 45 T, and in pulsed fields up to 65 T using a piezoresistive cantilever (SEIKO-PRC120). As the torque becomes substantial beyond the quantum limit, the resulting large deflection angle can drive both techniques out of their linear response regime. To correct for deviations from the magnetoresistance of the cantilever, field-sweeps of positive and negative polarity were averaged.

### Extraction of non-oscillatory background

**Figure 6:**
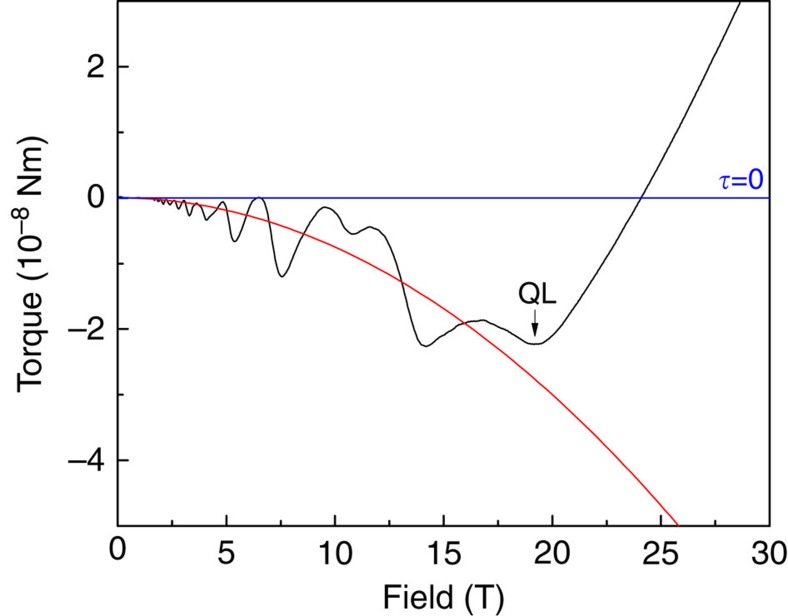
Extraction of the non-oscillatory torque. Example of the procedure used to separate the oscillatory component of the magnetic torque from the background, at an angle of 25° and a temperature of 4 K. The black line corresponds to the measured data, the red line to a parabolic fit and an arrow indicates the quantum limit field given by the last quantum oscillation. Clearly, the quantum limit also marks the torque anomaly associated with a sudden change in slope of the magnetic torque.

The sign reversal is a feature of the non-oscillatory component of the torque. On top of this, sizable quantum oscillations are commonly present close to the quantum limit of high-mobility, low carrier density semimetals. To extract the non-oscillatory background, a second-order polynomial was fitted through the data in the field range between zero and the quantum limit. This fit described the data sufficiently at all angles and evidently provides a good approximation for the non-oscillatory torque. Yet the sign reversal of the torque is independent of the background fitting and directly evident in the raw data: Even in the presence of strong quantum oscillations around 10 T-12 T, it is apparent that the non-oscillatory component of the torque is negative, and then changes sign after the quantum limit (see Fig. 6).

### Distinction between Dirac and Weyl fermions

To conceptually illustrate the potential utility of magnetic torque as a tool to diagnose Weyl semimetal materials, we explain how the quantum limit magnetization signals would differ for Dirac semimetals—which can be thought of as two opposite-chirality copies of a Weyl semimetal superposed in momentum space, but protected from mixing by crystal symmetry. The experimental results suggested that Dirac semimetal materials, Cd_3_As_2_ and Na_3_Bi, contain a pair of Dirac nodes whose massless nature is protected by a discrete (screw) rotation symmetry about their connecting axis.

**Figure 7:**
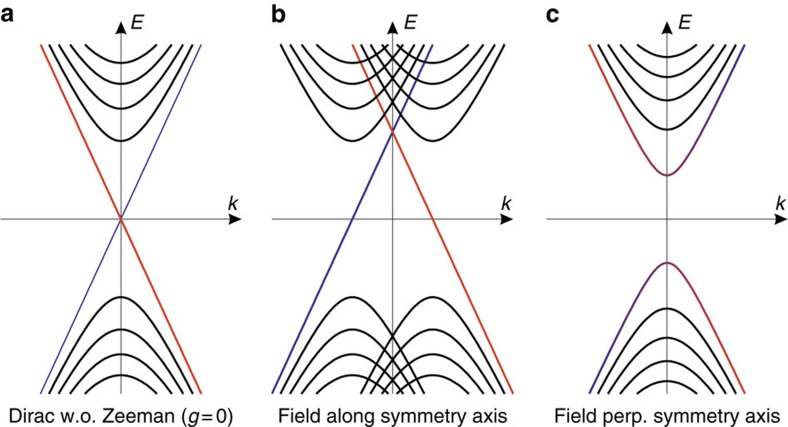
Landau levels for a Dirac semimetal. Schematic depiction of Landau level structure for a Dirac semimetal neglecting the Zeeman coupling (**a**) and including Zeeman coupling (**b**,**c**). For field along the high-symmetry (001) axis (**b**) the Dirac node splits into two opposite chirality Weyl nodes, each with a chiral *n*=0 Landau level whose energy is independent of field and gives paramagnetic response near the quantum limit. For field perpendicular to the high-symmetry axis (**c**) the field induces a gap in the *n*=0 Landau levels, giving rise to an ordinary diamagnetic response.

Due to the importance of crystal symmetry, the resulting magnetization signal from such a Dirac material is sensitive to the field orientation. For the fictitious case of a purely orbital magnetic field (that is, ignoring Zeeman coupling, Fig. 7a), a Dirac semimetal would behave as two independent copies of a Weyl semimetal, producing a magnetization reversal presented in the Discussion section. However, in the real materials a Zeeman coupling to the pseudo-spin degrees of freedom of the Dirac cone will be generated. The resulting magnetization depends strongly on the orientation of the applied field. For fields along the symmetry axis (Fig. 7b), the protecting symmetry is intact, and the Dirac cone splits into two Weyl nodes separated along the high-symmetry axis by momentum displacement ∼*H*. In this case, near the quantum limit, one expects to see a torque reversal near the quantum limit as in the Weyl material NbAs.

In contrast, applying a magnetic field perpendicular to the high symmetry axis (Fig. 7c) breaks the protecting crystal symmetry and induces a Dirac mass ∼*H*. In this case, the counter-propagating *n*=0 LLs for the Dirac node split in energy by ∼*H*, and behave more like ordinary massive LLs—that is, producing a diamagnetic response near the quantum limit. In this case, one would not observe a sign reversal in the torque near the quantum limit.

In contrast to the Weyl semimetal—where one expects a torque reversal near the quantum limit at any field angle, for a Dirac semimetal, one would instead observe a torque reversal that disappears as the field is tilted away from the high symmetry axis. In particular, sweeping the field orientation for fixed field strength in a Dirac semimetal would exhibit strong deviations from the sin2*θ* angle dependence expected in Weyl semimetals.

### Valence band contribution to magnetization

In this section we derive an approximate analytic expression for the valence band contribution to magnetization. Throughout, we work at zero temperature, and choose units such that *ħ*=*c*=*e*=1. We use the Landau band dispersion relation 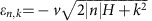, valid in the vicinity of the Weyl node. Here, *n* is a LL index and *k* is the momentum along the field. Each LL has extensive degeneracy 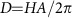, where *A* is the 2D area perpendicular to the applied field. We model a finite bandwidth by introducing a momentum cutoff Λ so that we restrict −Λ<k<Λ.

The ground-state magnetization per unit volume is given by 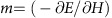, where *E* is the ground-state energy density:


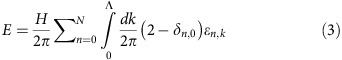


where *N* is the maximal LL index. We determine *N* by demanding that the density of valence electrons, 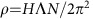, is fixed as a function of field, so that 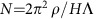.

Due to the sharp momentum space cutoff, *E* contains rapidly oscillating components with characteristic 1/*B* frequency ∼ρ/Λ, as well as a smooth background dependence on field, *H*. To isolate the smooth part of *E*, we use the Poisson resummation formula: 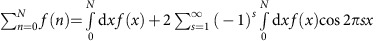. Applied to (2) with 
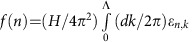
, the first term (s=0) produces the zero-field ground-state energy, which does not affect the magnetization, and can be dropped. We first perform the x-integration. Then, noting that experimentally accessible field scales are much smaller than valence bandwidth, *H*<<ρ/Λ, we can rescale 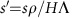, and evaluate the s-summation as a continuous integral. Lastly, dropping rapidly oscillatory factors 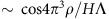, we obtain:


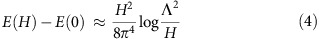


which, upon differentiation, gives the valence band contribution to magnetization quoted in the discussion.

We can understand the functional form of this result perturbatively as follows: the field *H* couples to the electrons via the vector potential *A*∼*H* × *r*∼*H*/*k*, which induces interband transitions between valence and conduction band states. This gives a second order in *H* correction to the energy 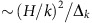, where 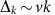 is the band-gap between conduction and valence bands, which vanishes at the Dirac point (*k*=0). Integrating this result over the valence band gives 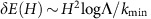. Here, the small momentum log-divergence of this perturbative expression is cutoff by the larger of the two quantities: 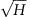, or the Fermi momentum of the conduction band (since inter-band corrections can occur only between occupied and unoccupied states).

### Data availability

The authors present all raw data used for the analysis in the manuscript.

## Additional information

**How to cite this article:** Moll P. J. W. *et al*. Magnetic torque anomaly in the quantum limit of Weyl semimetals. *Nat. Commun.* 7:12492 doi: 10.1038/ncomms12492 (2016).

## References

[b1] Vafek O. & Vishwanath A. Dirac fermions in solids—from high Tc cuprates and graphene to topological insulators and Weyl semimetals. Annu. Rev. Condens. Matter Phys. 5, 83–112 (2014).

[b2] Geim A. K. Graphene: status and prospects. Science 324, 1530–1534 (2009). 19541989 10.1126/science.1158877

[b3] Kane C. L. An insulator with a twist. Nat. Phys. 4, 348–349 (2008).

[b4] Wang Z., Weng H., Wu Q., Dai X. & Fang Z. Three-dimensional Dirac semimetal and quantum transport in Cd_3_As_2_. Phys. Rev. B 88, 125427 (2013).

[b5] Liu Z. K. . Topological Dirac semimetal, Na_3_Bi. Science 343, 864–867 (2014). 24436183 10.1126/science.1245085

[b6] Liu Z. K. . A stable three-dimensional topological Dirac semimetal Cd_3_As_2_. Nat. Mater. 13, 677–681 (2014). 24859642 10.1038/nmat3990

[b7] Borisenko S. . Experimental realization of a three-dimensional Dirac semimetal. Phys. Rev. Lett. 113, 027603 (2014). 25062235 10.1103/PhysRevLett.113.027603

[b8] Huang S.-M. . A Weyl Fermion semimetal with surface Fermi arcs in the transition metal monopnictide TaAs class. Nat. Commun. 6, 7373 (2015). 26067579 10.1038/ncomms8373 PMC4490374

[b9] Shekhar C. . Extremely large magnetoresistance and ultrahigh mobility in the topological Weyl semimetal candidate NbP. Nat. Phys. 11, 645–649 (2015).

[b10] Wehling T. O., Black-Schaffer A. M. & Balatsky A. V. Dirac materials. Adv. Phys. 63, 1–76 (2014).

[b11] Hosur P. & Qi X. Recent developments in transport phenomena in Weyl semimetals. Comptes Rendus Phys. 14, 857–870 (2013).

[b12] Novoselov K. S. . A roadmap for graphene. Nature 490, 192–200 (2012). 23060189 10.1038/nature11458

[b13] Zyuzin A. A. & Burkov A. A. Topological response in Weyl semimetals and the chiral anomaly. Phys. Rev. B 86, 115133 (2012).

[b14] Arnold F. . Negative magnetoresistance without well-defined chirality in the Weyl semimetal TaP. Nat. Commun. 7, 11615 (2016). 27186980 10.1038/ncomms11615 PMC4873626

[b15] Shoenberg D. Magnetic oscillations in metals Cambridge University Press (1984).

[b16] Berry M. V. Quantal phase factors accompanying adiabatic changes. Proc. R. Soc. A Math. Phys. Eng. Sci. 392, 45–57 (1984).

[b17] Zak J. Berrys phase for energy bands in solids. Phys. Rev. Lett. 62, 2747–2750 (1989). 10040078 10.1103/PhysRevLett.62.2747

[b18] Brignall N. L. The de Haas-van Alphen effect in n-InSb and n-InAs. J.Phys.C: Solid State Phys. 7, 4266–4276 (1974).

[b19] Wang Z. . Dirac semimetal and topological phase transitions in A3Bi (A=Na, K, Rb). Phys. Rev. B 85, 195320 (2012).

[b20] Koshino M. & Ando T. Anomalous orbital magnetism in Dirac-electron systems: role of pseudospin paramagnetism. Phys. Rev. B 81, 195431 (2010).

[b21] Mikitik G. P. & Sharlai Y. V. The phase of the de Haas-van Alphen oscillations, the Berry phase, and band-contact lines in metals. Low Temp. Phys. 33, 439–442 (2007).

[b22] Mikitik G. P. & Sharlai Y. V. Berry phase and de Haas-van Alphen effect in LaRhIn5. Phys. Rev. Lett. 93, 106403 (2004). 15447428 10.1103/PhysRevLett.93.106403

[b23] Goodrich R. G. . Magnetization in the ultraquantum limit. Phys. Rev. Lett. 89, 026401 (2002). 12097010 10.1103/PhysRevLett.89.026401

[b24] Ghimire N. J. . Magnetotransport of single crystalline NbAs. J. Phys. Condens. Matter 27, 152201 (2015). 25814484 10.1088/0953-8984/27/15/152201

[b25] Weng H., Fang C., Fang Z., Bernevig B. A. & Dai X. Weyl semimetal phase in noncentrosymmetric transition-metal monophosphides. Phys. Rev. X 5, 011029 (2015).

[b26] Luo Y. . Electron-hole compensation effect between topologically trivial electrons and nontrivial holes in NbAs. Phys. Rev. B 92, 205134 (2015).

[b27] Ali M. N. . Large, non-saturating magnetoresistance in WTe 2. Nature 514, 205–208 (2014). 25219849 10.1038/nature13763

[b28] Furuseth S. & Kjekshus A. On the arsenides and antimonides of niobium. Acta Chem. Scand. 18, 1180–1195 (1964).

